# Low-Burden Digital Phenotyping of Affective Risk: Positive Emoji Usage, Speech Rate, and Sleep Relate to College Student Mental Health

**DOI:** 10.21203/rs.3.rs-9226835/v1

**Published:** 2026-04-16

**Authors:** Robert D. Henry, Karishma Singh, Grace Mooney Anderson, Luke Goldammer, Anya Kapitula, Cassie Morse, Jacqueline Rivera, Violet Wasilevich, Shreeya Behera, York Li, Jeremy Jamieson, Tara Maddala

**Affiliations:** 1 TSET Health Promotion Research Center, University of Oklahoma Health Campus, Tulsa, OK 74135; 2 Department of Biostatistics & Epidemiology, University of Oklahoma Health Campus, Oklahoma City, OK 73104; 3 Department of Psychology, University of Rochester, Rochester, NY 14627; 4 Department of Psychology, Hope College, Holland, MI 49422; 5 Department of Statistics and Data Science, Northwestern University, Evanston, IL 60208; 6 School of Information Sciences (iSchool), University of Illinois Urbana-Champaign, Champaign, IL, 61820; 7 Pandora Bio Inc, Sunnyvale, CA 94087

**Keywords:** mental health, young adulthood, depression, anxiety, digital markers

## Abstract

Mental health problems tied to negative affective experiences are common among emerging adults, yet conventional symptom questionnaires provide only distal snapshots of dynamic affective processes that unfold in daily life. Using the novel Meet Pandora smartphone application, we examined how everyday affective experiences and passive markers relate to depressive symptoms (PHQ), anxiety symptoms (GAD), and psychological flourishing in college students (*N* = 120; *N*_*obsercations*_ (PHQ/GAD) = 372, *N*_*obsercations*_ (flourishing) = 792). We tested three data channels (self-report, voice-derived features, and behavioral indicators) and disaggregated within-person fluctuations from between-person differences using multilevel models. Across outcomes, the proportion of positive emojis selected to reflect one’s affective state emerged as the most consistent signal. On days when participants used a higher-than-usual proportion of positive emojis, they reported lower depression and anxiety, and individuals with higher average positive emoji use reported lower symptoms and higher flourishing. Positive emoji use also moderated change over time in flourishing, such that flourishing increased across the study period only among participants with relatively high positive emoji proportions. In contrast, passive features showed more selective associations. Speech rate (words per minute) was linked to lower symptom burden and higher flourishing in some models, and longer average sleep duration was associated with lower anxiety and higher flourishing. Overall, results highlight the value of separating within- from between-person effects when linking digital markers to mental health and suggest that low-burden indicators of positive affect may be especially informative for scalable temporal monitoring of affective risk and wellbeing in young adults.

Mental health challenges among young adults have become an increasingly problematic public health concern. National data from the Healthy Minds Study (2021–2022) indicate that approximately 44% of college students screen positively for depression, 34% for anxiety, and nearly half report frequent loneliness (Eisenberg et al., 2023). These patterns are particularly concerning given that roughly 75% of lifetime mental disorders emerge before 24 (Kessler et al., 2005; Solmi et al., 2022), making late adolescence and emerging adulthood a critical window for detecting affective risk and promoting well-being.

From an affective science perspective, anxiety and depression are understood as disorders with emotional underpinnings that unfold over time rather than as static symptom profiles. Negative affect (encompassing anxiety, distress, and shame) is robustly associated with adverse mental health outcomes, whereas positive affect reflects the presence of pleasurable emotional states such as joy, contentment, and hope, and is linked to improved mental and behavioral health (Fortenberry et al., 2020; Stringer, 2020). Affective experiences vary both between and within individuals across time, and these fluctuations are shaped by regulatory processes that determine how emotions are generated, maintained, and resolved (Crowell et al., 2020). Emotion dysregulation, characterized by excessive reactivity, volatility, or blunted emotional responding, has been implicated as a core mechanism underlying both anxiety and depression (Mennin et al., 2005; Gonçalves et al., 2019; Vlisides-Henry et al., 2021).

## Conventional symptom measures as windows into affective experiences

Widely used screening instruments such as the Patient Health Questionnaire (PHQ) and the Generalized Anxiety Disorder scale (GAD) continue to serve as clinical benchmarks for assessing depression and anxiety. Although these measures were not designed explicitly as affective instruments, their items index emotional, cognitive, and somatic experiences that unfold in daily life (e.g., nervousness, worry, fatigue, anhedonia). Thus, PHQ and GAD scores may be understood as distal summaries of more proximal affective processes; for instance, daily affective states (e.g., anxiety, sadness) correlate strongly with PHQ and GAD scores at both within- and between-person levels (Lakhtakia et al., 2024; Nexha et al., 2024). Though within-person affective dynamics provide information not captured by between-person differences alone, evidence also suggests that within-person *reliability* of affective indicators is often modest, even when between-person reliability is strong (Diest et al., 2024). Altogether, prior literature highlights a need for careful disaggregation of within- and between-person variance of PHQ and GAD when understanding these measures in the context of affective dynamics.

## Everyday life, affect, and emotion regulation

A growing body of research demonstrates that everyday emotional states and behaviors prospectively predict affective risk (i.e., heightened and labile negative emotion). Loneliness and experiential avoidance, which can be understood as maladaptive emotion regulation strategies, have been shown to predict increases in anxiety, depression, and general psychological distress over time (Moroz & Dunkley, 2019; Richardson et al., 2017). Sleep disturbances represent another critical pathway: deviations from one’s typical sleep duration or quality predict next-day anxiety, while elevated anxiety reciprocally predicts poorer sleep the following night (Chan et al., 2022). During the COVID-19 pandemic, self-isolation, often adopted as a short-term coping strategy, was associated with higher depressive symptoms, highlighting how regulatory behaviors that provide immediate relief may exacerbate longer-term affective risk (Narita et al., 2023). Collectively, these findings emphasize that affective risk emerges from dynamic, context-sensitive processes unfolding in daily life. Traditional retrospective questionnaires, administered weeks or months apart, are limited in their ability to capture these processes. As a result, there is increasing interest in methods that assess affect “in the wild.”

## Measuring affect in daily life using EMA combined with passive sensing

Ecological momentary assessment (EMA) and passive sensing technologies offer powerful tools for capturing emotional experiences and behaviors as they occur. EMA enables repeated sampling of affective states in real time, reducing recall bias and increasing ecological validity. Passive sensing complements EMA by providing continuous behavioral and contextual data (e.g., movement, sleep, speech) that may reflect underlying regulatory processes (Bettis et al., 2021; Kaplan et al., 2023). Yet, the richness of these data raises important questions about parsimony and interpretability. Evidence suggests that although multiple sensor streams are often necessary to capture mental well-being, predictors should be selected based on theory rather than breadth alone (Das Swain et al., 2022; Vlisides-Henry et al., 2021). Null findings are therefore informative: for example, Culbreth et al. (2025) found that certain passive indicators predicted negative symptoms but not depressive or positive affect, clarifying the boundaries of what passive data might capture.

## Young adulthood as a critical risk period

Young adulthood and the transition to college often coincides with shifts in sleep patterns, social networks, autonomy, and stress exposure, all of which can destabilize emotion regulation. Over 80% of university students report poor sleep quality, which is associated with elevated anxiety and depressive symptoms (Whitehead & Horton, 2025). These disruptions may give rise to daily affective fluctuations that precede more enduring symptom trajectories. Despite elevated need, many college students at highest risk do not seek professional support. Large-scale studies indicate that students experiencing severe distress or suicidality often report low likelihood of future help-seeking and face greater socioeconomic and academic challenges (Nash et al., 2017; Rastogi et al., 2024). Digital mental health assessment tools that leverage mobile technologies may help bridge this gap by providing accessible, real-time monitoring and tailored resources. However, their effectiveness depends on understanding which signals meaningfully reflect affective risk.

## Present study

The present study examines how daily affective experiences and passive behavioral markers relate to depression, anxiety, and psychological flourishing in a large sample of college students. Using data collected via the Meet Pandora smartphone application, we investigated associations across three data channels (self-report, voice-derived biomarkers, and behavioral indicators) while disaggregating within- from between-person effects using multilevel modeling. By examining both total symptom scores and everyday affect, this work clarifies how conventional mental health measures reflect dynamic emotional processes and identifies which digital markers provide meaningful information about affective risk in daily life.

## Method

### Participants

All methods were approved by the University of Rochester Institutional Review Board (STUDY00008705). Participants were undergraduate students enrolled at a northeast U.S. university and were recruited through a university study participation portal. Students participated during the Spring 2025 semester (*N*=120). See Table 1 for demographic information. On average, participants were enrolled in the study for more than 50 days, and the recruitment period was intended to last 12 weeks in the initial pilot and a minimum of 8 weeks thereafter (with the option to continue participation). Participants were aged 18–26 years (*M*=21, *SD*=1.8) and the sample was demographically diverse (though female students were overrepresented). Of the full sample, *n* = 27 (22.7%) reported a clinically significant baseline PHQ-8 score, and *n* = 29 (24.4%) reported a clinically significant baseline GAD-7 score. Furthermore, *n* = 39 (32.8%) self-reported a diagnosis of a psychological disorder.

### Self-Report Measures

#### Anxiety Symptoms

Anxiety was measured using the Generalized Anxiety Disorder-7 (GAD-7; Spitzer et al., 2006). Participants completed this measure at baseline, end of week 4, and end of week 8 (approximately every 30 days). The scale asks participants to report how often, over the past two weeks, they have been bothered by symptoms such as “*feeling nercous, anxious, or on edge*,” using a 4-point response scale from 0 (*not at all*) to 3 (*nearly ecery day*) (α = .86).

#### Depressive Symptoms

Depression symptoms were evaluated using the Patient Health Questionnaire–8 (PHQ-8; Kroenke et al., 2009). Participants completed this measure at baseline, end of week 4, and end of week 8 (approximately every 30 days). The scale asks participants to report how often they have been bothered by symptoms such as “*little interest or pleasure in doing things*,” using a 4-point response scale with options 0 (*not at all*) to 3 (*nearly ecery day*) (α = .86).

#### Psychological Flourishing

The extent to which participants felt successful in domains such as relationships, purpose, optimism, and self-esteem was measured weekly using the Flourishing Scale (Diener et al., 2010). An example item includes “*My social relationships are supportice and rewarding*.” The scale includes eight questions asking participants to report the extent to which they agree with statements reflecting psychological well-being, using a 7-point response scale from 1 (*Strongly disagree*) to 7 (*Strongly agree*) (α = .91).

#### Acceptance, Loneliness, and Feelings of Burden

Single-item measures of perceived acceptance, loneliness, and feelings of burden were administered weekly to participants. Participants reported how accepted they felt by their friend group, how often they felt lonely, and the extent to which they felt they were a burden to friends or family. Loneliness and burden items were reverse scored, meaning that higher scores on any item indicated more positive social well-being. Response scales ranged from 1 (*Very slightly or not at all*) to 5 (*Extremely*) for loneliness and burden, and from 1 (*Not at all accepted*) to 5 (*Strongly accepted*) for perceived acceptance.

#### Daily Affect via Emoji

**Figure 1:**
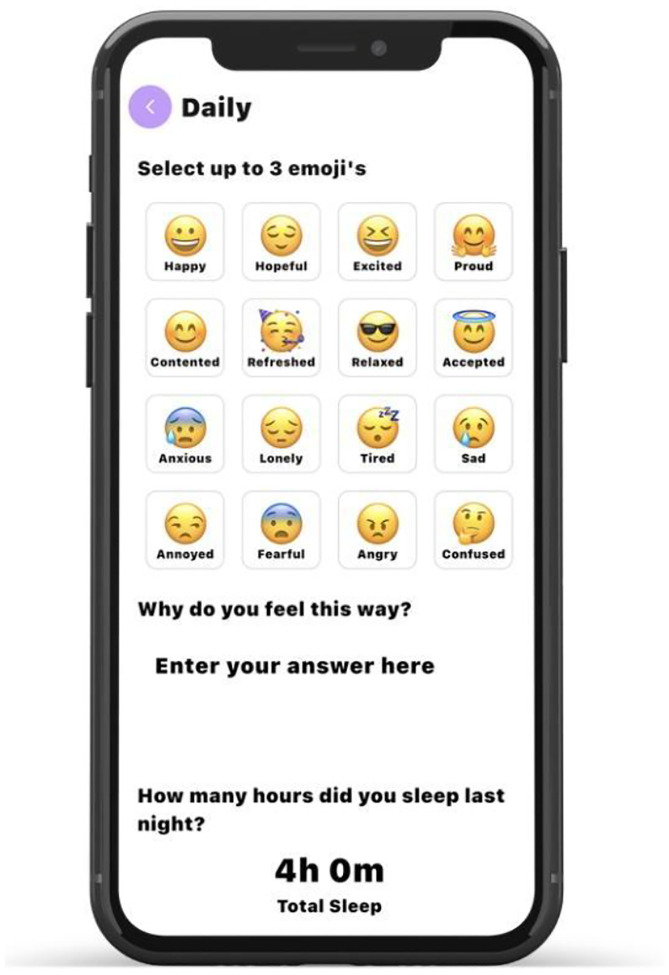
Example Screen Depicting How Participants Reported Daily Emoji Affect *Note*. The above image depicts the screen participants saw when reporting affect via emojis. Sixteen emojis were available for participants to select and participants could select up to three in any instance; first eight options were positive while the latter eight were negative.

Daily emotional experience was assessed through a brief emoji check-in. Each day, participants were asked to select up to three (but at least one) emojis that best represented how they felt at that time. Emojis were displayed alongside corresponding affective labels to support clarity of interpretation (see Figure 1). Available options (and their positive/negative categorization) were Accepted (positive), Angry (negative), Annoyed (negative), Anxious(negative), Confused (negative), Contented (positive), Excited (positive), Fearful (negative), Happy (positive), Hopeful (positive), Lonely (negative), Proud (positive), Refreshed (positive), Relaxed (positive), Sad (negative), and Tired (negative). Responses were recorded each day. The number of positively-valenced emojis reported were then summed and divided by the total number of emojis reported in a day to derive the daily proportion of positive emoji affect.

### Passive Measures

#### Voice Biomarkers

Voice-derived markers were calculated from participant speech recordings to capture daily variation in vocal and linguistic markers. Approximately 3 times per week, students were prompted to voice journal by responding to one of 20 questions, such as “Talk about a conversation you had with a friend in the last week.” Questions were either positive or neutral in nature and intended to elicit a response between 30 seconds and two minutes. Participants could choose the valence (positive or neutral) of the question. Approximately 47.8% of the total 2,241 vocal samples were based on positive questions/prompts. Of the biomarkers relevant for this study, question valence was significantly related to only word count (*p* = .001), such that positive questions were related to more words spoken. Four features were extracted from each vocal sample: words per minute, Shannon entropy, utterance count, and mean pitch. These indicators served as proxies for speech rate, variability, verbal output, and vocal tone, respectively. Features were processed at the daily level and later separated into within-person and between-person components for multilevel modeling.

#### Behavioral Markers

GPS data were used to estimate movement dynamics and activity patterns each day. Passive location sampling recorded coordinates throughout the day, from which daily steps and number of distinct places visited were derived. These indicators were processed as continuous behavioral signals and decomposed into within-person and between-person values for multilevel modeling. Higher values reflected greater movement, and more locations visited. Number of meals and sleep durations were self-reported daily.

#### Procedures

Eligible participants were university students between the ages of 18 and 31 who resided in the United States, could read and communicate fluently in English, and had access to a mobile device capable of running the Meet Pandora application (iOS 12 or later or Android OS 14 or later). Meet Pandora collected self-report, geolocation, and biometric inputs and securely transmitted all information under the HIPAA-compliant data handling protocols. Although biometric information could be paired with wearable devices when available, the study did not require the use of a wearable and did not restrict participation to any specific device type.

**Figure 2:**
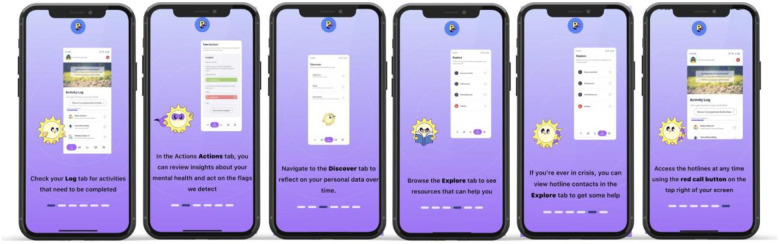
Meet Pandora App Overview/Introduction to Meet Pandora App *Note*. The images above depict screenshots of the introductory screen participants saw when onboarding to the Meet Pandora app.

Once students reviewed the study overview and provided electronic consent, they were guided through the app’s onboarding pages where they acknowledged the privacy notice and consented to terms of use (Figure 2). During enrollment, users were invited to enable permissions for passive data streams, including location tracking, microphone and camera access, and general phone analytics; however, no information about specific websites or applications visited was recorded to maintain privacy. Participants first completed an intake survey assessing demographic characteristics and individual preferences. Subsequent assessments occurred on staggered timeframes. Brief daily check-ins and weekly surveys captured mood, behavior, and emotional experiences. Stress and flourishing were assessed each week, and anxiety and depression were evaluated on a monthly cycle. GPS sampling ran passively in the background, typically at fifteen-minute intervals when the participant was stationary and approximately one-minute intervals during movement.

The application generated visual feedback displays summarizing patterns and changes in users’ emotional states, behavior, and well-being. When notable shifts emerged in indicators such as anxiety, depression, loneliness, acceptance, or substance use, the system notified participants with supportive nudges. Each participant also received a personalized set of resource recommendations at onboarding, and these suggestions updated automatically when patterns in self-report or passive data signaled meaningful changes.

##### Treatment of Missing Data.

There were 372 non-missing PHQ and GAD observations in total, and 792 non-missing flourishing observations. To evaluate the plausibility of missing-at-random (MAR) assumptions, we conducted missingness checks. First, we examined within-wave attrition by modeling the probability that PHQ/GAD/flourishing outcomes were missing on a given wave/day as a function of observed time-varying and person-level covariates, allowing for clustering within participants. We ran a series of multilevel logistic regressions, first predicting the 20 missing PHQ/GAD cells from variables used in the final models and found at least marginal associations between missingness and day number, vocal pitch, words per minute, and sleep (*ps* < .09). The same was done for flourishing. Only six missing flourishing scores appeared in the dataset; thus, we did not run within-wave missingness models for flourishing.

Second, we examined predictors of attrition by modeling whether participants completed all scheduled symptom assessments (i.e., 3 or more) using person-level covariates. We again used multilevel logistic models, predicting attrition (i.e., completing fewer than 3 assessments) with predictors used in models. The following features associated with higher odds of attrition (*p*s < .086): loneliness, words per minute, sleep, and sexuality (identifying as non-heterosexual). A similar multilevel Poisson model was done predicting the number of flourishing observations, and words per minute and sleep tended to predict higher odds of attrition (*p*s < .066). Based on these data checks, we assumed MAR was plausible.

##### Data Analysis Plan.

We estimated a series of multilevel models in R (v.4.3.1) using the *lme4* package (v.1.1–34; Bates et al., 2015) to examine associations between self-reported, behavioral, and speech indicators and mental health outcomes. Given the intensive longitudinal nature of the predictor data, time-varying predictors were aggregated using a rolling approach. Specifically, for each outcome assessment, predictor values were averaged across all available days leading up to and including the day of the assessment. For instance, if PHQ was assessed at baseline (day 0), day 30, and day 60, predictors such as loneliness were averaged across day 0 for the baseline assessment, across days 1 through 30 for the second assessment, and across days 31 through 60 for the third assessment.

All models included disaggregated within- (Level-1) and between-person (Level-2) components of time-varying predictors. Within-person predictors were person-mean centered, and between-person predictors were operationalized as each individual’s person-level mean. This modeling approach allowed us to disaggregate within-person fluctuations from stable between-person differences for each predictor (Curran & Bauer, 2011). Time in study (day) was included as a Level-1 predictor in all models. Gender, self-reported childhood adverse experiences (ACEs), sexual orientation, and first-year student status were included as Level-2 covariates. For brevity, the effects of these covariates are not reported. Analyses were conducted separately by data channel. That is, self-report predictors of interest were examined in their own models, followed by separate models for vocal biomarkers and behavior features. This channel-specific modeling strategy reduced multicollinearity, facilitated interpretability across distinct data streams, and allowed for clearer comparison of effects across measurement modalities. The generalized multivariable model is depicted below in mixed equation form:

$$Y_{ti}=\gamma_{00}+\sum_{k=1}^{K}\gamma_{k}^{\left(B\right)}{\overset{-}{X}}_{k,i}+\beta_{li}{Day}_{ti}+\sum_{k=1}^{K}\beta_{k}^{\left(w\right)}\left(X_{k,ti}-{\overset{-}{X}}_{k,i}\right)+u_{0i}+u_{0i}e_{ti}$$

where *Y*_*ti*_ denotes the outcome variable (i.e., total PHQ, GAD, or flourishing) for a given person *i* on a given day *t*; *γ*_*00*_ denotes fixed (grand-mean) intercept; *Day*_ti_ is the study day for a person *i* on day *t* and *β*_*1i*_ is its fixed effect; *X*_*k,ti*_ captures the value of each time-varying predictor *k* for a person *i* on day *t* (up to *K* total predictors); ${\overset{-}{X}}_{k,i}$ person *i*’s mean for time-varying predictor *k*; $\gamma_{k}^{\left(B\right)}$ refers to the between-person effect of between mean-levels of predictor *k* on the outcome; $\beta_{k}^{\left(w\right)}$ is the within-person association between day-to-day deviations in predictor *k* from a person’s own mean and the outcome; *u*_*0i*_ is the random intercept; and *e*_*ti*_ denotes within-person residual. Flourishing models included random slopes for time.

Results are organized by outcome (depressive symptoms, anxiety symptoms, and flourishing) and by data channel (self-report, voice-derived biomarkers, and GPS-derived behavioral markers). Given the exploratory goal of identifying candidate digital markers and our relatively small sample size, inferential tests were not treated as definitive “discoveries.” However, to reduce the risk of false discoveries and to avoid over-interpretation of our findings, in addition to raw-unit fixed effects (*b*s) with unadjusted p-values (*p*_u_), we calculated Benjamini-Hochberg (BH) corrected p-values (*p*_BH_) and reported each for corresponding significant *p*_u_ values.

## Results

### Depressive Symptoms

#### Self-Report Channel Model

First, day in the study did not significantly predict PHQ scores (*p*_u_ = .09, *p*_BH_ = .17). At the within-person level, students reported lower depressive symptoms on occasions when they used a higher-than-usual proportion of positive emojis, though this effect did not survive the BH correction (*b* = −2.79, *p*_u_ = .026, *p*_BH_ = .086). No other within-person emotional states significantly predicted PHQ scores based on unadjusted p-values (*p*s_u_ > .055, *p*s_BH_ > .14). At the between-person level, students who, on average, reported a greater proportion of positive emojis exhibited significantly lower overall PHQ scores (*b* = −6.46, *p*_u_ < .001, *p*_BH_ < .001). Higher mean loneliness (*b* = 1.40, *p*_u_ < .001, *p*_u_ = .003), and higher mean avoidance (*b* = 1.12, *p*_u_ = .005, *p*_BH_ = .022), were also associated with greater depressive symptoms. Mean perceived unacceptance, feeling like a burden, and overall self-evaluative feelings were not significantly related to PHQ scores (*p*s_u_ > .15, *p*s_BH_ > .24).

#### Voice Channel Model

Day in the study did not significantly predict PHQ scores (*p*_u_ = .89, *p*_BH_ = .93). At the within-person level, higher-than-usual utterance count was (marginally) associated with lower depressive symptoms (*b* = −0.34, *p*_u_ = .029, *p*_BH_ = .209). Similarly, at the between-person level, higher average words per minute was marginally associated with lower PHQ scores (*b* = −0.04, *p*_u_ = .047, *p*_BH_ = .209). All other within-person and between-person vocal features were not significantly related to PHQ scores (*p*s_u_ > .20, *p*s_BH_ > .60).

#### Behavioral Channel Model

Day in the study did not predict PHQ scores (*p*_u_ = .54, *p*_BH_ = .61). The only significant predictor was a within-person effect of meals, such that on days when participants logged more meals than usual, they reported (marginally) higher depressive symptoms (*b* = 1.14, *p*_u_ = .047, *p*_BH_ = .42). All other within-person and between-person behavioral features, including steps, places visited, and hours of sleep, were not significantly related to PHQ scores (*p*s_u_ > .24, *p*s_BH_ > .47).

### Anxiety Symptoms

#### Self-Report Channel Model

Day in the study did not significantly predict GAD scores (*p*_u_ = .43, *p*_BH_ = .55). At the within-person level, higher-than-usual proportions of positive emojis were associated with lower anxiety symptoms (*b* = −3.30, *p*_u_ = .004, *p*_BH_ = .042). Within-person loneliness was also positively associated (marginal after correction) with GAD scores (*b* = 0.56, *p*_u_ = .020, *p*_BH_ = .065). Other within-person emotional states were not significantly related to anxiety (*p*s_u_ > .37, *p*s_BH_ > .47). At the between-person level, students who reported a higher average proportion of positive emojis exhibited (marginal after correction) lower overall GAD scores (*b* = −4.15, *p*_u_ = .017, *p*_BH_ = .065). Higher mean avoidance was associated with greater anxiety (*b* = 1.21, *p*_u_ = .006, *p*_BH_ = .042). No other between-person predictors were significantly related to GAD scores (*p*s_u_ > .17, *p*s_BH_ > .46).

#### Voice Channel Model

At the between-person level, higher average words per minute was associated with lower GAD scores (*b* = −0.06, *p*_u_ = .004, *p*_BH_ = .033). This effect suggests that for every 100 words per minute spoken an individual had, on average, associated with GAD scores that were 6 pts lower than others. No other vocal features significantly predicted anxiety symptoms (*p*s_u_ > .18, *p*s_BH_ > .64).

#### Behavioral Channel Model

Higher average hours of sleep associated with lower GAD scores at the between-person level, but the effect no longer reached significance after correction and is thus not interpreted (*b* = −1.11, *p*_u_ = .036, *p*_BH_ = .33). No other behavioral predictors were significantly related to anxiety symptoms (*p*s_u_ > .14, *p*s_BH_ > .42).

### Psychological Flourishing

#### Self-Report Channel Model

Flourishing models included a random slope for day in the study, allowing trajectories of flourishing to vary across students. For this model, given our use of random slopes for time, we chose to include an exploratory interaction term between positive emoji usage and days in the study. A significant interaction emerged (*b* = 0.22, *p*_u_ = .020, *p*_BH_ = .041) and simple slopes analyses indicated that flourishing increased over time only when participants reported relatively high proportions of positive emojis (90th percentile of within-person positive emoji usage: *b* = 0.05, *p* = .006)^1^. The association between time and flourishing was not significant at average or low levels (10th percentile) of positive emojis (*p*s_u_ > .066, *p*s_BH_ > .), suggesting that increases in positive affect may amplify gains in flourishing over time.

At the within-person level, feeling unaccepted (*b* = −0.96, *p*_u_ < .001, *p*_BH_ < .001), avoidance (*b* = −0.47, *p*_u_ = .015, *p*_BH_ = .036), and feeling like a burden (*b* = −0.67, *p*_u_ < .001, *p*_BH_ = .003) were each associated with lower flourishing. Within-person positive mood, loneliness, and time were not significant main effects (*p*s > .061). At the between-person level, higher average proportions of positive emojis were associated with greater flourishing (*b* = 14.10, *p*_u_ < .001, *p*_BH_ < .001), whereas higher mean unacceptance (*b* = −3.89, *p*_u_ < .001, *p*_BH_ < .001), and loneliness (*b* = −1.52, *p*_u_ = .027, *p*_BH_ = .047), were associated with lower flourishing. No other between-person predictors were significant (*p*s > .07).

#### Voice Channel Model

Day in the study did not significantly predict flourishing scores (*p*_u_ = .19). At the within-person level, participants reported higher flourishing on days when they spoke more words per minute than was typical for them; however, this effect was no longer significant after BH correction (*b* = 0.04, *p*_u_ = .045, *p*_BH_ = .20). Similarly, at the between-person level, participants who, on average, spoke more words per minute reported marginally higher overall flourishing (*b* = 0.07, *p*_u_ = .031, *p*_BH_ = .20). All other within-person and between-person vocal features were not significantly related to flourishing (*p*s > .081).

#### Behavioral Channel Model

Day in the study did not significantly predict flourishing scores (*p*_u_ = .23). At the within-person level, participants reported higher flourishing on days when they consumed more meals than their personal average (*b* = 3.06, *p*_u_ < .001, *p*_BH_ < .001), and when they took more steps than usual (*b* = 0.0005, *p*_u_ = .007, *p*_BH_ = .02). These effects indicate that one additional meal compared to a person’s averaged predicted flourishing scores that were 3.06 pts higher; additionally, every 100 more steps a person took (relative to their mean) associated with 0.05 pts of flourishing increase. Within-person number of places visited and sleep duration were not significantly associated with flourishing (*p*s > .14). At the between-person level, longer average sleep duration was associated with higher flourishing (*b* = 2.78, *p*_u_ = .001, *p*_BH_ = .005). No other between-person behavioral predictors were significantly related to flourishing (*p*s > .14).

## Discussion

This study examined how daily self-reported affective experiences (emojis), voice-derived features and behavioral indicators relate to depressive symptoms, anxiety symptoms, and psychological flourishing. By disaggregating within-person fluctuations from between-person differences, our findings clarify when “in-the-moment” changes in affect and behavior covary with mental health (state-like associations) versus when stable individual differences in everyday affective patterns and routines map onto well-being (trait-like associations).

### Self-report findings: Positive emoji usage as a mental health marker

The proportion of positive emojis used appeared to be a consistent marker of well-being, whereas most passive/behavioral features showed either sparse associations or outcome-specific patterns. Thus, emoji usage may represent a modern and “lightweight” approach to examining daily affect and may provide useful measurement of psychological distress and well-being that may enhance efficiency and specificity compared to traditional self-report measures given how common emoji-based affective communication has become in recent years (Erle et al., 2022; Fischer & Herbert, 2021; Mulligan et al., 2022; Pine et al., 2024).

Across depression and anxiety models, higher-than-usual positive emoji use predicted lower symptoms at the within-person level, and higher average positive emoji use predicted lower symptoms at the between-person level. These convergent findings suggest that positive affective expression in daily life may operate both as a momentary buffer (days characterized by more positive affect coincide with fewer symptoms) and as a stable protective factor (higher positive affect individuals show lower symptom burden). This supports not only the detrimental role of heightened negative affect, but also the protective, broadening, and restorative functions of positive affect displays in emotion regulation processes (Battaglini et al., 2022; Swerdlow et al., 2022). The positive emoji effects emerged even when other self-reported emotional states were largely non-significant within-person predictors of symptoms, suggesting that this relatively simple behavioral measure may capture meaningful variance in daily affective experience. This finding is also potentially relevant for social processes, as increased displays of positive affect can help beneficial emotion regulation strategies “spill over” from one person to another in times of stress (Oveis et al., 2020).

With respect to flourishing, people who reported a high proportion of positive emoji use tended to have higher flourishing over the course of the study. Contrastingly, those with average or low use of positive emojis had no association between time and flourishing. This moderation effect aligns with gain amplification or “positive upward spiral” theories on human flourishing, which state that individuals who capitalize on opportunities for positive emotion more easily grow in positive affect over time (Fredrickson & Joiner, 2018; Fredrickson & Losada, 2005; Yeager et al., 2022). This finding also suggests that, over time, not everyone “regresses to the mean”; indeed, flourishing growth depends on affective context. For instance, a recent latent profile analysis found that people with the highest flourishing tended to have affect that was low in variability and slow to change dynamically (Mohammed et al., 2025). Further research is needed to better understand and categorize the causal dynamics of young adults’ moment-to-moment flourishing, as well how those data might be leveraged to build psychological interventions to promote flourishing in the face of omnipresent life stressors (see Jamieson et al., 2018 for a review)

Beyond emojis, overall (between-person) loneliness and avoidance associated with higher depressive symptoms, while avoidance only weakly predicted anxiety. At the within-person level, loneliness predicted anxiety (but not depression). The lack of robust within-person associations is likely due to the limited number of within-person observations. Yet, findings support prior work highlighting the strong, socially-mediated impact on depressive symptoms—more so than anxious symptoms—in young adults (Yang et al., 2023; Yuan et al., 2022). Our models showed several robust within-person associations between self-report measures and flourishing (i.e., unacceptance, avoidance), and acceptance and loneliness also associated between-person. These results underscore the social nature of human flourishing (e.g., VanderWeele, 2017; Vidal et al., 2022).

### Detection of mental health signal among vocal and behavioral biomarkers

Vocal biomarkers and behavioral measures exhibited few associations with depression, anxiety, and flourishing, and many of the effects that emerged did not survive Benjamini-Hochberg correction. Within-person utterances and between-person words per minute associated weakly with lower depressive symptoms, and the opposite was true for within-person number of meals (note that words per minute associated more robustly with lower anxiety). Prior studies have found that vocal biomarkers (e.g., pitch, loudness/magnitude) may differentiate those with major depression from healthy controls (Menne et al., 2024; Zhao et al., 2022). However, given that our sample consisted of relatively healthy, functioning college students, our results suggest that how much a person speaks may predict their risk for a pending depressive episode (e.g., fewer utterances could be a sign of prodromal withdrawal). More longitudinal research will need to be done to verify this claim, though, as it is beyond the scope of the current design.

With respect to flourishing, none of the vocal biomarkers survived corrections for false discovery. Within the behavioral marker model, consuming more meals than usual and taking more steps than usual robustly predicted more flourishing, and people who slept more had higher flourishing. These contrasting findings from the PHQ and GAD models further support the notion that flourishing and mental wellbeing in general is not merely the “opposite” of anxiety and depression. This reinforces the value of our affective dynamics approach: collapsing across levels could obscure mechanisms and mislead intervention targets. By examining both mental *health* (flourishing) and psychopathology *risk* across active and passive measures, we tapped into the affective dynamics of young adults.

### Limitations and future directions

Several limitations shape our interpretation. First, all effects are correlational, meaning that causality cannot be inferred. Future work should gather more repeated measures to assess more complex temporal associations as well as designing experimental approaches to induce affective experiences in digital contexts (e.g., Lee et al., 2020). Second, our vocal and behavioral measures likely contain substantial noise (literal and theoretical) and may be sensitive to compliance, platform differences, and situational constraints. For instance, participants voluntarily opted into providing vocal samples, creating a self-selection bias. Additionally, participants were not necessarily instructed to keep their phones on their person throughout the study, meaning that GPS-derived indicators could be misleading. Finally, null findings do not imply absence of effects, and additional feature engineering (especially for mobility) and multimodal fusion approaches could reveal higher-order patterns not detectable in our single-channel models. Our sample was also relatively homogeneous, which may have blurred our ability to detect GPS-derived differences (e.g., most participants lived on a campus, so “locations visited” might not have been an informative marker in hindsight).

## Conclusion

In a sample of college students, daily positive affective expression via emojis emerged as a robust marker of lower depression and anxiety and higher flourishing. Vocal and behavioral markers provided selective signals that were weaker in magnitude (particularly speech rate), underscoring the importance of theory-driven marker selection. Overall, these findings support an affective dynamics view of mental health in young adulthood and suggest that scalable, low-burden indicators of positive affect (emoji usage) may be especially valuable for digital monitoring, and affective processes may be important targets for intervention aimed at reducing risk and promoting well-being in young adults.

## Figures and Tables

**Figure 3:**
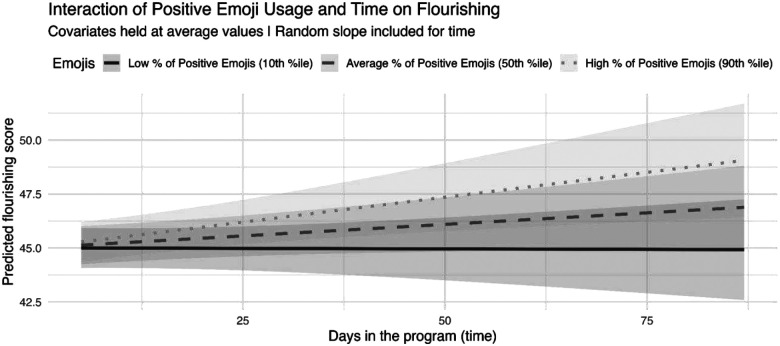
*Note*. This plot depicts simple slopes of the association between days since enrollment and flourishing at low (10th percentile), mean (50th), and high (90th) of the proportion of positive emojis used. Only the high proportion slope was significantly different from zero (*b* = 0.05, *p* = .006).

**Table 1:** Participant Demographics

Characteristic	*n* (%) or Statistic
*Gender*	
Male	16 (13.3%)
Female	99 (82.5%)
Other	5 (4.2%)
*Sexual Orientation*	
Heterosexual	86 (71.7%)
LGBTQ+	33 (27.5%)
Other	1 (0.8%)
*Neurodivergent*	
Yes	39 (32.5%)
No	78 (65.0%)
Did not respond / Missing	3 (2.5%)
*First-Generation Student*	
Yes	21 (17.5%)
No	99 (82.5%)
*International Student*	
Yes	24 (20.0%)
No	96 (80.0%)
*Religion*	
Atheist	17 (14.2%)
Unaffiliated	32 (26.7%)
Jewish	7 (5.8%)
Muslim	6 (5.0%)
Hindu	6 (5.0%)
Christian	29 (24.2%)
Other	23 (19.2%)
*Race*	
Mixed	62 (51.7%)
White	40 (33.3%)
Asian	0 (0.0%)
Hispanic, Latino/a/x or Spanish origin	8 (6.7%)
Other	10 (8.3%)
*Time in Study (days)*	
Mean (*SD*)	51.3 (17.4)
Median	55.0
Range	0–89

*Note*. Percentages are based on the total sample (*N* = 120).

## Data Availability

Data are available upon request.

## Footnotes

1 The interaction effects of time ✕ positive emoji usage predicting PHQ and GAD were non-significant and thus were not included in the primary results (*ps* > .13).
